# Exploring the dietary choices of Chinese women living with breast cancer in Vancouver, Canada

**DOI:** 10.1007/s00520-020-05666-9

**Published:** 2020-08-09

**Authors:** Brenda Ng, Gwen E. Chapman, Ryna Levy-Milne, Lynda G. Balneaves

**Affiliations:** 1 Vancouver, Canada; 2 College of Social and Applied Human Sciences, University of Guelph, Guelph, ON Canada; 3 College of Nursing, Rady Faculty of Health Sciences, University of Manitoba, Winnipeg, MB Canada

**Keywords:** Breast cancer, Women, Chinese, Diet, Qualitative research

## Abstract

**Purpose:**

To explore how Chinese Canadian patients with breast cancer make dietary choices and to understand their nutritional information needs in order to inform oncology healthcare providers about provision of optimal supportive care for this population.

**Methods:**

Using interpretive description methodology, semi-structured interviews were conducted with first- and second-generation Chinese Canadian women aged 41–73 years living in Vancouver, Canada, who were diagnosed with breast cancer within the last 5 years. A follow-up focus group was held to validate emergent themes.

**Results:**

Nineteen women were interviewed; 6 participated in the focus group. Their accounts of dietary experiences following diagnosis focused on three areas: dietary change (including desired and implemented changes that participants believed would benefit their health), facilitators and barriers to dietary change, and information and resource needs. Dietary changes reported included avoiding or consuming greater amounts of certain foods, and taking traditional Chinese medicine (TCM) and natural health products. Barriers to desired dietary change included the interplay between food preferences and family and social life, work-life balance, and cost and availability of specialty foods. Support from family members, however, facilitated participants’ consumption of more whole and natural foods after their cancer diagnosis. Participants obtained food and nutrition information from a variety of sources but had difficulty determining the reliability and accuracy of information. They requested timely, credible, culturally-relevant, and easily accessible dietary information.

**Conclusions:**

Oncology healthcare providers would benefit from increased understanding of the dietary practices, including TCM, of Chinese women living with breast cancer. To facilitate communication and improve quality of care, healthcare professionals should provide credible and culturally relevant diet-related information in a variety of forms.

## Introduction

Breast cancer is the most frequently diagnosed cancer in women in Canada [1]. Between 41 and 57% of Canadian women diagnosed with breast cancer report dietary changes after cancer diagnosis [2, 3], with decreases in animal food products (77%) and increases in fruit and vegetable intake (72%) being the most prominent. Women provide various reasons for these changes, including reducing the risk of recurrence and improving overall health for themselves and their families [2–4]. However, some express confusion when making food choices as they received conflicting advice on diet and nutrition from healthcare professionals and their social network.

Immigrant Chinese women in Western countries who are diagnosed with breast cancer may face particular challenges when making food choices as they navigate between Western medical messaging about diet and cancer and Chinese cultural understandings of the complex influences of food on health and illness [5]. The general belief that all foods have either “hot” or “cold” properties is very common among Chinese people, including those who have migrated to Western countries [6]. According to the concept of dietary therapy in traditional Chinese medicine (TCM), hot (yang) foods tend to warm the body while cold (yin) foods dispel body heat [7]. Optimal health is a result of the equilibrium between hot and cold energy, whereas illness results from a shift in balance incurred by extreme diets, lifestyle factors, climate, and emotional stress. Such imbalance may lead to loss of vitality and malfunctioning of internal organs, which contributes to systemic illness over time [5]. Traditional Chinese dietary beliefs also hold that certain foods “cause” diseases while other foods can help “fight” illness [7, 8]. Recognizing and designating the “poisonous-tonic” nature of food have helped guide dietary choices of Chinese people for generations.

Studies in Hong Kong, USA, and Canada have found that Chinese cancer patients integrate cultural beliefs about food and health to prevent cancer recurrence and promote health [5, 7, 9–11]. Many use TCM and dietary therapy to complement conventional medical treatments [5, 7, 12, 13]. There is evidence that these patients experience confusion about dietary choices and dissatisfaction with the reliability and specificity of information on effects of food on cancer [11, 12, 14]. However, very little research has been conducted to understand the specific food experiences and dietary information needs among Chinese women living with breast cancer in Canada. This information is crucial for oncology healthcare providers to ensure that they are able to provide optimal supportive care for this population.

We, therefore, conducted a qualitative study to explore how Chinese Canadian women with breast cancer make dietary choices and to understand their nutritional information needs. Vancouver, Canada, was an appropriate setting for the study as it has a large Chinese population with diverse linguistic and cultural backgrounds.

## Methods

This study utilized interpretive description, a qualitative research approach that asks questions that explore human phenomenon within a specific context, analyzes data to identify meanings, characteristics, and patterns, and produces theoretically sound and clinically useful findings [15]. The study was approved by the Behavioural Research Ethics Board of the University of British Columbia. Written informed consent was obtained from all participants.

We used purposive sampling to recruit first-generation (i.e. immigrant) and second-generation (i.e. born in Canada) Chinese Canadian women living in Vancouver who were Cantonese, Mandarin, or English speaking and diagnosed with breast cancer within the last 5 years. We deliberately sought a diverse sample in terms of immigration and language because this is characteristic of the Vancouver Chinese Canadian community and we wanted to understand any differences in experiences and needs within this group. Chinese and English language posters and recruitment cards were displayed at outpatient cancer care clinics at the cancer centre serving the Vancouver region, provided to patients by cancer centre oncologists, or circulated at the centre’s Chinese cancer support group sessions. Recruitment continued until saturation (or redundancy of information) was reached [16].

Semi-structured interviews were conducted using an interview guide based on the interpretive description approach [15]. Participants were asked to describe their typical dietary choices before and after their cancer diagnosis, their dietary beliefs, the dietary decision-making process used, their nutritional information needs, and suggestions for information resources they would like available. All interviews were conducted by the first author (BN), who is proficient in English, Cantonese, and Mandarin, in the language(s) preferred by the participant. Interviews lasted for about an hour and were digitally recorded and transcribed verbatim by BN, who translated interviews conducted in Cantonese and Mandarin to English at the time of transcription.

NVivo 10 software was used to facilitate data analysis, which included (1) manually coding and decontextualizing the data; (2) sorting and categorizing the codes; and (3) developing topics and interpretive schema [17]. BN was the primary analyst; the research team reviewed early transcripts together to discuss coding schemes and reach consensus on the codes, then continued to meet regularly throughout the data collection and analysis process to discuss transcripts, codes, and emerging themes.

Following initial analysis of the interview data, all participants were invited to a follow-up focus group to validate the preliminary findings, enhance the depth of the inquiry, identify contextual and individual circumstances of the dietary decision-making process, and ensure the trustworthiness of the findings [18]. Three members of the research team (BN, GEC, and RLM) attended. The session was facilitated by BN who presented the themes developed during preliminary analysis and translated as needed between English (the main language used during the focus group) and Cantonese. Participants were asked to discuss in their preferred language ways in which the findings were similar to and different from their experience and identify anything they thought was missing. The focus group was digitally recorded and notes were taken during the session. The data collected during the focus group were organized and integrated into the findings during the final analysis and writing.

## Results

Nineteen first- and second-generation Chinese Canadian women were recruited (Table 1) and interviewed. Participants ranged in age from 41 to 73 years. The 14 first-generation Chinese Canadian participants had lived in Canada from 8 to 40 years; 7 were Cantonese-speaking, 6 were Mandarin-speaking, and 1 was English-speaking. The remaining five women were second-generation Chinese Canadians, predominantly English-speaking, whose Cantonese-speaking parents migrated from Southern China to Vancouver. The study sample included women at different phases of cancer treatment, from newly diagnosed patients receiving active treatment to those who were close to 5 years post-diagnosis and had completed all treatment. Fifteen women were married and most had children. All but three had some post-secondary education (Table 1). Six of the participants attended the focus group. All women were first-generation participants and understood some English though only one regularly spoke English at home. Of the other five, four spoke Cantonese at home and one spoke Mandarin.

**Table 1:** Demographic characteristics of study participants (*n* = 19)

Characteristics		*N*
Age (years)	40–49	7
	50–59	6
	60–69	5
	70 or above	1
Language spoken at home	English	6
	Cantonese	7
	Mandarin	6
Place of birth	Vancouver	5
	China	4
	Hong Kong	6
	Taiwan	4
Time since diagnosis	12 months or less	5
	13–24 months	8
	25–60 months	6
Marital status	Single	2
	Married	15
	Divorced	2
Highest education level	Elementary school completed	1
	High school completed	2
	College/diploma	5
	Undergraduate	10
	Post-graduate	1

Study participants’ accounts of their dietary experiences following their breast cancer diagnosis focused on three areas: dietary change (including desired and implemented changes that participants believed would benefit their health), facilitators and barriers to dietary change, and information and resource needs. Focus group participants confirmed that their experiences in making dietary choices before and after cancer diagnosis were consistent with the 3 key themes identified in the preliminary analyses of interview data. They also provided additional examples in support of these themes. Interview and focus group data are integrated in the presentation of data below.

### Dietary changes

Most participants stated that they had made significant changes to their diet following their breast cancer diagnosis. Women reported three main types of dietary changes: avoiding specific foods, consuming more of certain foods, and taking TCM and nutritional supplements.

Almost all participants indicated that they had reduced consumption of animal protein, including chicken, beef, and salmon. Some indicated they were concerned about antibiotics and growth hormone residue found in meat and dairy products, such as a 61-year-old woman who said “I used to have milk, soymilk, toast and sometimes oatmeal for breakfast but I am not drinking milk now as some people said that milk has hormones.” A number of Cantonese-speaking and second-generation participants mentioned that they avoided certain foods, such as duck and seafood, which they described as “duk,” which means poisonous in Cantonese. Some participants believed these foods might exert adverse effects on their cancer and aggravate inflammation. In contrast, Mandarin-speaking participants generally did not mention any food that they believed was “poisonous.” Some participants talked about avoiding meat because of acid-alkali concerns and wanting to balance the pH in their blood. Other types of food participants avoided included soy products, deep-fried and barbequed foods, processed foods, and sugar.

I like deep-fried pork chop, beef, beef noodle soup. I don’t eliminate them completely but I would go for… beef noodle soup maybe only once a month now. I love deep-fried food but staying away from them… deep-fried meats and tofu.

Participants also described increasing consumption of specific types of foods, including fresh fruits, vegetables, beans, and lentils. About half of the group indicated that they preferred buying organic produce because of concerns about residues of chemical fertilizers and pesticides found in regular produce. Most participants stated that they preferred traditional Chinese foods after treatments, such as congee (traditional Chinese plain rice porridge), rice noodles in fish soup, and, for Cantonese-speaking participants, traditional Chinese homemade soups. One first-generation participant, who spoke predominantly English and had moved to Vancouver when she was 8 years old, indicated that she usually had a mix of Asian and Western cuisines in her everyday diet. However, during treatment, she found herself avoiding Western food that made her feel sick and craving traditional “comfort” food, such as congee, which reduced her nausea. A second-generation woman also pointed out that she preferred traditional Chinese food like congee or traditional soups when she was sick because “It’s just soothing and comforting.”

The third type of diet-related changes reported by participants was increased use of TCM and nutritional supplements. Several women were taking TCM in the form of capsules (e.g. Cordyceps or Reishi mushrooms) while those who had consulted TCM doctors consumed Chinese medicine in the form of decoction, which is a preparation of herbs, botanicals (usually dried), and/or animal ingredients placed in water, boiled until the volume is markedly reduced, and the residues strained off. Cordyceps or Reishi mushrooms, consumed in either capsules or soups, were used to increase strength and energy after surgery and as an adjuvant to chemotherapy. A number of participants also started taking nutritional supplements, such as grapeseed extract, fish oil, calcium, and antioxidant drinks, after their diagnosis.

Participants provided a variety of reasons for their dietary changes, including maintaining a healthy body weight, reducing risk of breast cancer recurrence, alleviating symptoms resulting from conventional treatments, helping family members adopt healthier diets, and wanting to live longer. For example, one woman said: “I gained weight during treatment and I know that maintaining a healthy BMI is important to help reduce recurrence so I am trying to be a little bit healthier.” Another woman shared: “I really don’t want any of my family members to have cancer… also I think by consuming a healthier diet, it’s also good for cardiovascular health.”

Participants’ stories regarding their experiences with diet and dietary change following their cancer diagnosis revealed beliefs as well as uncertainties about traditional Chinese health concepts. First-generation participants often talked about practical aspects of food choice and preparation in ways that reflected traditional concepts of ying/yang and hot/cold. For example, one woman talked about preparing a traditional Lo For Tong soup and said: “I think these soups are good for our health as lamb soups warm us up, warm up our hands and legs. And so does chicken soup - this is a Chinese tradition.” Another woman said, “Some vegetables are of cold-nature, for example mustard greens. It is very cold (nature) so putting in some ginger can help balance the coldness.” These concepts were mostly mentioned by Cantonese-speaking women, though one Mandarin speaker from Mainland China talked about making soups to balance her “cold” body. One Taiwanese woman mentioned this concept but said she did not believe in it.

Despite this practical knowledge, most women did not display in-depth theoretical knowledge of these concepts and indicated that they learned about TCM and dietary therapy from their family members and/or TCM practitioners. As well, many participants expressed uncertainty about the validity and rationale underlying these dietary beliefs and practices. These included a few second-generation participants, who consumed traditional soups prepared by their mothers out of appreciation for their concern and effort. Other participants received conflicting messages from healthcare professionals about the effects and/or benefits of drinking traditional soups after a cancer diagnosis: “I noticed that different doctors have different opinions, so for now I will still drink Lo For Tong but not drinking too much.”

### Barriers and facilitators to dietary changes desired and implemented by participants

The participants experienced various obstacles when attempting to implement their post-diagnosis dietary changes. Specifically, this included (1) interplay between food preferences and family and social life; (2) work-life balance; and (3) cost and availability of specialty foods. Some of these obstacles, however, also acted as facilitators that promoted healthful eating for several participants.

Participants’ dietary changes sometimes involved trying to eat less of the foods that they and their family members and friends enjoyed eating, and/or consuming a greater amount of foods that were less preferred. Adhering to dietary goals was easier when family and friends were supportive of those goals, especially when they tried to eat in the same way as the patient. For example, one woman reported that her husband supported her by preparing food with her and eating less meat together. Another woman discussed how her friends, who were also experiencing health issues, were trying to “eat more fish, more fruits and veggies, more steaming; less deep-frying, less drinking, less sweets…that kind of thing.” However, other women found family members were resistant and wanted to eat the foods that they were familiar with. One woman shared: “I am having a tough time convincing my family” to eat less red meat and more beans and lentils. Social and festive occasions were particularly challenging for the women, because food items were selected based on taste, traditions, and meanings rather than health effects: “It’s not just about feeling full or satisfying our appetite… eating with friends and family is for socializing… like for Chinese New Year, we would still eat turnip cake with preserved meats, Chinese sausages and dried shrimps… I still eat these with my family and friends and feel very happy… if I had to totally eliminate these foods from my diet, it would definitely affect my emotional well being.”

Many participants indicated that they were consuming less packaged and processed food because of the perceived health benefits of having more homemade, whole foods in their diets. However, some found it challenging to allocate time for meal planning, grocery shopping, and cooking. One participant with a busy work schedule said, “I wish I had the time. If I could buy my food from somebody who will make everything healthy, I would eat healthy, but it really has to be convenient.”

Finally, the cost of specialty foods such as organic produce, meat and poultry, and Cordyceps mushrooms was identified as a challenge by some women. While participants were generally able to find these foods in Vancouver, the higher price compared with conventional foods was sometimes prohibitive, “When it comes to buying organic, it’s half and half ‘cause sometimes it’s just too expensive. I can’t afford it. Sometimes Costco has a good deal on organic chicken so I will buy it; otherwise, I will just use regular chicken.”

### Information and resource needs

Participants sought and obtained food and nutrition information from a variety of sources, including family, friends, fellow cancer survivors, internet, social media, oncologists, dietitians, an integrative supportive cancer care clinic, library books, TCM practitioners, and family physicians. Some participants had received information and attended a nutrition class delivered by dietitians at their local cancer treatment centre. Given the range of information sources, not surprisingly, many women reported receiving conflicting information. This often related to traditional Chinese practices; while first-generation Chinese Canadian participants usually followed traditional practices without concern, second-generation participants found the advice they were receiving from their mothers contradicted their own, more Western, beliefs. When they proactively searched for information to verify the advice they were receiving, they had difficulty determining what information was reliable and accurate. As a result, participants expressed a need for timely, credible, culturally relevant, easily accessible information.

Participants requested information specific for different stages of the cancer trajectory. Women receiving active treatment generally felt weak and some experienced difficulty eating, including nausea and vomiting. These women expressed the need for information to help plan their meals, cope with the symptoms associated with cancer treatments, and regain energy. In contrast, women in the recovery phase indicated they would like to learn about food and nutrition to help reduce the risk of cancer recurrence and promote overall health.

While most second-generation women indicated that current nutrition resources were culturally adequate for them, first-generation women noted that information often did not address ingredients and preparation methods that they were familiar with:

This booklet was good but all the ingredients listed were based on Western diet, beans, hummus…not commonly consumed by Chinese and not usually found in our diet. It would be helpful to use this booklet as the basis and modify, to develop a booklet that talks about Chinese ethnic food, Chinese cooking methods and recipes, the seasonings, spices.

First-generation women also noted that they were interested in group education sessions presented by registered dietitians who were familiar with Chinese dietary practices and ideally conducted in Cantonese and/or Mandarin.

## Discussion

This study explored dietary choices and information needs of Chinese Canadian women living with breast cancer in Vancouver, Canada. Findings showed that participants made dietary changes following diagnosis that were influenced by a number of factors, including Western and/or traditional Chinese principles, and that facilitators and barriers to making these changes included family and social/lifestyle factors. Participants were seeking credible and culturally relevant diet-related information to promote health as well as to prevent the recurrence of breast cancer.

Some dietary changes that participants made were similar to those reported by women diagnosed with breast cancer in the general population [2, 4, 19]. The most common of these, decreasing animal protein and increasing vegetable, fruit, and legume intake, are consistent with evidence-based recommendations from the American Institute of Cancer Research/World Cancer Research Fund global diet and cancer report [20]. However, participants also talked about using traditional Chinese foods and/or TCM post-diagnosis to support the management of cancer. They believed that diet was implicated in their cancer occurrence, recovery, and recurrence, which resonates with the findings from other studies with immigrant Chinese cancer patients in other countries. For example, Liu, Sun, and Louie [21] found that the Chinese American women with cancer they surveyed used TCM to manage specific cancer-related symptoms. Payne, Seymour, Chapman, and Holloway [22] also found that older Chinese people living in the UK discussed the role of food in health and illness, including cancer, as being “risky,” “therapeutic,” or “comforting.”

Where our findings differ is with the heterogeneity found among participants with difference linguistic and/or regional backgrounds. Specifically, the women in our sample who were from Taiwan did not practice and/or believe in TCM concepts, and mostly relied on media and medical professionals for diet-related information after cancer diagnosis. In contrast, the Mandarin-speaking participants from Mainland China did believe in TCM concepts and consumed traditional foods for health maintenance, and Cantonese speakers from Hong Kong and China were the participants who talked about consuming traditional homemade soups and herbal decoctions as a means to protecting and restoring their health.

There were also differences between first- and second-generation participants. Second-generation participants, all of whom were born to Cantonese-speaking parents, generally did not hold traditional dietary beliefs but learned some TCM concepts and the practice of consuming traditional soups and herbal decoctions for health maintenance from their mothers. Despite their uncertainties, they consumed the soups as a token of appreciation for the effort their mothers put into preparing the soups. There were also differences in information-seeking behaviours and information needs, especially with regard to the use of TCM and dietary therapy after diagnosis, between first- and second-generation participants.

Our study was limited by its small sample size and in that only 6 of the 19 interview participants returned for the focus group. Because of this, we cannot make strong claims about regional and generational differences. However, the findings suggest the need for further research to more fully explore heterogeneity in beliefs, practices, and information needs within immigrant Chinese communities. It is important for healthcare providers to be aware of this heterogeneity and not assume that all patients from a particular ethnic background will have similar needs or make similar choices.

With regard to facilitators and barriers to dietary change, family support or resistance was a significant influence. Participants stated that it was easier to adhere to dietary changes if family and friends were supportive, particularly when they followed a similar diet. Other researchers have noted that food is often used to express love and care in Chinese culture, especially among family members [23]. Cost was identified as a barrier to dietary change for some participants, particularly in relation to purchasing organic foods. Other studies of Chinese immigrant cancer patients have not identified the consumption of organic foods as a priority. This may reflect the culture and environment of the Vancouver community, where there is a strong discourse regarding environmentally safe agricultural practices and availability of organic products.

Studies conducted with Chinese patients in other countries, including China, have identified the need for culturally sensitive and linguistically appropriate resources about food and dietary guidelines [11, 12, 24–26]. This is similar to the findings of the present study. However, our participants’ proactive seeking of information contrasts the findings of a study conducted in Hong Kong [5] where it was noted that participants did not search for answers to their questions about food and diet, even when they encountered uncertainties. These findings may reflect how healthcare providers are viewed as authoritative figures within Chinese culture and Chinese patients’ hesitancy or fear of asking questions related to their health, as well as the belief that doctors were unwilling to discuss diet-related matters. Our participants’ proactive seeking of diet-related information might be attributed to their high level of educational achievement and a culture-shift in this community. The women in our study were willing and capable of communicating with healthcare providers about their diet and breast cancer.

Finally, in the present study, some participants indicated that they did not inform their oncologists or dietitians about their use of TCM as they felt these professionals did not understand TCM and might ask them to stop using it, similar to what was reported by Xu et al. [27]. To facilitate communication and ensure safety, healthcare providers should become more aware of the use of TCM among cancer patients, encourage them to ask questions about diet, TCM, and dietary therapy at various stages of the cancer trajectory, and provide accessible and culturally appropriate platforms to address their information needs, such as group classes, cooking lessons, and printed material.

## References

[1] Canadian Cancer Society (2019) Breast cancer statistics. http://www.cancer.ca/en/cancer-information/cancer-type/breast/statistics/?region=on. Accessed November 21, 2019

[2] Beagan BL, Chapman GE (2004). Eating after breast cancer: influences on women’s actions. J Nutr Educ Behav.

[3] Maunsell E, Drolet M, Brisson J, Robert J, Deschênes L (2002). Dietary change after breast cancer: extent predictors, and relation with psychological distress. J Clin Oncol.

[4] Adams C, Glanville NT (2005). The meaning of food to breast cancer survivors. Can J Diet Pract Res.

[5] Simpson PB (2003). Family beliefs about diet and traditional Chinese medicine for Hong Kong women with breast cancer. Oncol Nurs Forum.

[6] Lee MM, Shen JM (2008). Dietary patterns using traditional Chinese medicine principles in epidemiological studies. Asia Pac J Clin Nutr.

[7] Koo LC (1984). The use of food to treat and prevent disease in Chinese culture. Soc Sci Med.

[8] Mok E, Martinson I (2000). Empowerment of Chinese patients with cancer through self-help groups in Hong Kong. Cancer Nurs.

[9] Ashing-Giwa KT, Padilla G, Tejero J, Kraemer J, Wright K, Coscarelli A, Clayton S, Williams I, Hills D (2004). Understanding the breast cancer experience of women: a qualitative study of African American, Asian American, Latina and Caucasian cancer survivors. Psycho-Oncology.

[10] Chiu L, Balneaves L, Barroetavena MC, Doll R, Leis A (2006). Use of complementary and alternative medicine by Chinese individuals living with cancer in British Columbia. J Complement Integr Med.

[11] Leng J, Lee T, Sarpel U, Lau J, Li Y, Cheng C, Chang M, Gany F (2012). Identifying the informational and psychosocial needs of Chinese immigrant cancer patients: a focus group study. Support Care Cancer.

[12] Leng J, Lee T, Li Y, Stern C, Chen MH, Winkel G, Gany F (2014). Support needs of Chinese immigrant cancer patients. Support Care Cancer.

[13] Wang S, Windsor C, Yates P (2012). The process and patterns of combining the use of TCM and Western Medicine in Taiwanese people with cancer. J Nurs Educ Pract.

[14] Bell K, Lee J, Ristovski-Slijepcevic S (2009). Perceptions of food and eating among Chinese patients with cancer. Cancer Nurs.

[15] Thorne S, Reimer-Kirkham S, MacDonald-Emes J (1997). Interpretive description: a noncategorical qualitative alternative for developing nursing knowledge. Res Nurs Health.

[16] Glaser BG, Strauss AL (1967). The discovery of grounded theory: strategies for qualitative research.

[17] Thorne SE (2008). Interpretive description.

[18] Lambert SD, Loiselle CG (2008). Combining individual interviews and focus group to enhance data richness. J Adv Nurs.

[19] Salminen EK, Lagstrom HK, Heikkia SP, Salminen SJ (2000). Does breast cancer change patients’ dietary habits?. Eur J Clin Nutr.

[20] American Institute for Cancer Research/World Cancer Research Fund report (2018) Diet, nutrition, physical activity, and cancer: a global perspective. The third expert report. https://www.wcrf.org/dietandcancer. Accessed November 21, 2019

[21] Liu S, Sun Y, Louie W (2015). Symptom distress and its association with traditional Chinese medicine use in Chinese American women with cancer. Oncol Nurs Forum.

[22] Payne SA, Seymour JE, Chapman A, Holloway M (2008). Older Chinese people’s views on food: implications for supportive cancer care. Ethn Health.

[23] Wright LM, Watson WL, Bell JM (1996). Beliefs: the heart and healing in families and illness.

[24] Kwok C, White K (2011). Cultural and linguistic isolation: the breast cancer experience of Chinese-Australian women – a qualitative study. Contemp Nurse.

[25] Lee S, Chen L, Ma GX, Fang CY, Ma YO, Scully L (2013). Challenges and needs of Chinese and Korean American breast cancer survivors: in-depth interviews. N Am J Med Sci (Boston).

[26] Simpson PB (2003). Hong Kong families and breast cancer: beliefs and adaptation strategies. Psycho-Oncology.

[27] Xu W, Towers AD, Li P, Collet JP (2006). Traditional Chinese medicine in cancer care: perspectives and experiences of patients and professionals in China. Eur J Cancer Care.

